# Research advances in hypertriglyceridemia-induced acute pancreatitis

**DOI:** 10.3389/fmed.2026.1829495

**Published:** 2026-08-25

**Authors:** Yongxia Cai, Xizhu Huang

**Affiliations:** Department of Emergency Medicine, Sir Run Run Shaw Hospital, Zhejiang University School of Medicine, Hangzhou, China

**Keywords:** acute pancreatitis, hemofiltration, hypertriglyceridemia, plasma exchange, therapy

## Abstract

Hypertriglyceridemia-induced acute pancreatitis (HTG-AP), a significant subtype of acute pancreatitis (AP), is witnessing a rising global incidence and is associated with a poorer clinical prognosis compared to AP of other etiologies. This review outlines the fundamental theories, clinical characteristics, diagnostic modalities, therapeutic strategies, and future research directions concerning HTG-AP, with a particular emphasis on recent advancements in molecular biology, precision medicine, and therapeutic technologies. Epidemiological data reveal that patients with HTG-AP exhibit significantly higher rates of severe disease (46.9%) and multiple organ dysfunction syndrome (MODS) compared to those with AP of other causes; furthermore, concomitant biliary diseases or alcohol consumption exacerbate disease severity. Current evidence indicates that the pathogenesis of HTG-AP involves multifaceted mechanisms, including oxidative stress, activation of inflammatory signaling pathways, and intestinal barrier dysfunction. Notably, the crosstalk between the JAK2/P38MAPK signaling pathway and oxidative stress represents a pivotal mechanism. In terms of diagnosis, metabolomics has identified specific biomarkers such as phosphatidylethanolamine (PE), demonstrating a diagnostic area under the curve (AUC) of up to 0.962. Regarding treatment, the combination of plasma exchange and hemofiltration can rapidly reduce triglyceride (TG) levels (by up to 69.57%); however, the trade-off between cost and efficacy warrants careful consideration. Future research should prioritize the development of innovative diagnostic tools, the optimization of personalized therapeutic strategies, and the construction of comprehensive prevention systems to enhance the overall management of HTG-AP.

## Introduction

Acute pancreatitis (AP) is an acute chemical inflammation caused by multiple etiologies that lead to the activation of pancreatic enzymes within the pancreas, resulting in autodigestion, edema, hemorrhage, and even necrosis of the pancreatic tissue. Its precipitating factors include biliary diseases, alcohol consumption and dietary habits, hypertriglyceridemia, abdominal trauma, specific medications, and viral infections. Hypertriglyceridemic acute pancreatitis (HTG-AP) is acute pancreatitis induced by significantly elevated serum triglyceride levels. It currently ranks as the second leading cause of AP, following cholelithiasis. The diagnostic threshold is typically a serum triglyceride (TG) level ≥11.3 mmol/L. Alternatively, a diagnosis can be confirmed if the TG level is between 5.65 and 11.3 mmol/L with turbid, chylous serum (resembling milk or lard), after excluding other causes like gallstones and alcohol use.

The epidemiological landscape of hypertriglyceridemia-induced acute pancreatitis (HTG-AP) is characterized by distinct geographical disparities and specific demographic patterns. Data from multicenter cohort studies reveal that HTG-AP accounts for 33.75% of all acute pancreatitis (AP) cases, with a marked upward trend in incidence rising from 14.3% in 2001 to 35.5% in 2016 (1, 2). Compared to biliary AP, patients with HTG-AP present at a significantly younger age (mean age: 41 vs. 53 years) but exhibit a substantially higher severity profile. The rate of severe disease in HTG-AP is 46.9%, compared to only 17.6% in biliary AP, representing a 2.3-fold increased risk of developing multiple organ dysfunction syndrome (MODS) (3). Regarding gender distribution, males constitute the majority of HTG-AP cases (60.83%). This male predominance is even more pronounced in patients with combined alcohol-induced and hyperlipidemic AP (AHAP), where males account for 96.9% of cases (1, 4). Although rare, HTG-AP during pregnancy is associated with a fulminant clinical course. Hypertriglyceridemia is the underlying etiology in 50% of all pregnancy-associated AP cases. Among these, 35% progress to severe disease, resulting in a fetal mortality rate of 5% (5). HTG-AP demonstrates a significantly higher recurrence rate compared to AP of other etiologies. Approximately 64.8% of patients experience a recurrence within one year. Notably, recurrent cases are associated with inadequate lipid control post-discharge; triglyceride (TG) levels at one month after discharge remain elevated at 3.7 mmol/L in the recurrent group, compared to 2.0 mmol/L in the non-recurrent group (6). The clinical severity and complexity of HTG-AP translate into a substantial healthcare burden. Patients with HTG-AP have a significantly prolonged hospital stay (mean: 33 days vs. 24 days for biliary AP) and incur medical costs that are 1.8 times higher (3, 7).

The pathogenesis of HTG-AP results from the interplay between genetic susceptibility and environmental factors. Genetically, defects in the lipoprotein lipase (LPL) gene represent the most common monogenic etiology; for instance, the LPL p.His210Leu mutation can lead to a complete loss of enzymatic activity, causing serum TG levels to surge to 1,450 mg/dl and precipitating AP (8). Furthermore, the apolipoprotein E (ApoE) 3/2 genotype increases the risk of HTG-AP during pregnancy by reducing LPL activity (9). Among environmental factors, a diet high in sugars and fats, alcohol consumption, and certain medications (e.g., tamoxifen) are primary precipitants. Patients with combined alcohol-induced and hyperlipidemic AP exhibit a weekly alcohol intake of 140 g (compared to 80 g in pure alcohol-induced AP) and present with more severe disease, evidenced by a higher mean Modified CT Severity Index (MCTSI) score (8 vs. 5 in pure HLAP) (4, 10). Hormonal fluctuations during pregnancy also exacerbate lipid metabolism disorders; TG levels can increase three-fold after 28 weeks of gestation, with 20% of pregnant women exceeding 1,000 mg/dl (5). Additionally, obesity (BMI ≥ 30 kg/ m^2^) and diabetes mellitus aggravate hypertriglyceridemia through insulin resistance, increasing the risk of HTG-AP by 2.5-fold (11). Notably, a synergistic effect exists between genetic and environmental factors: patients carrying heterozygous LPL mutations who are concurrently obese face a 4.2-fold increased risk of developing HTG-AP (8).

**Figure 1:**
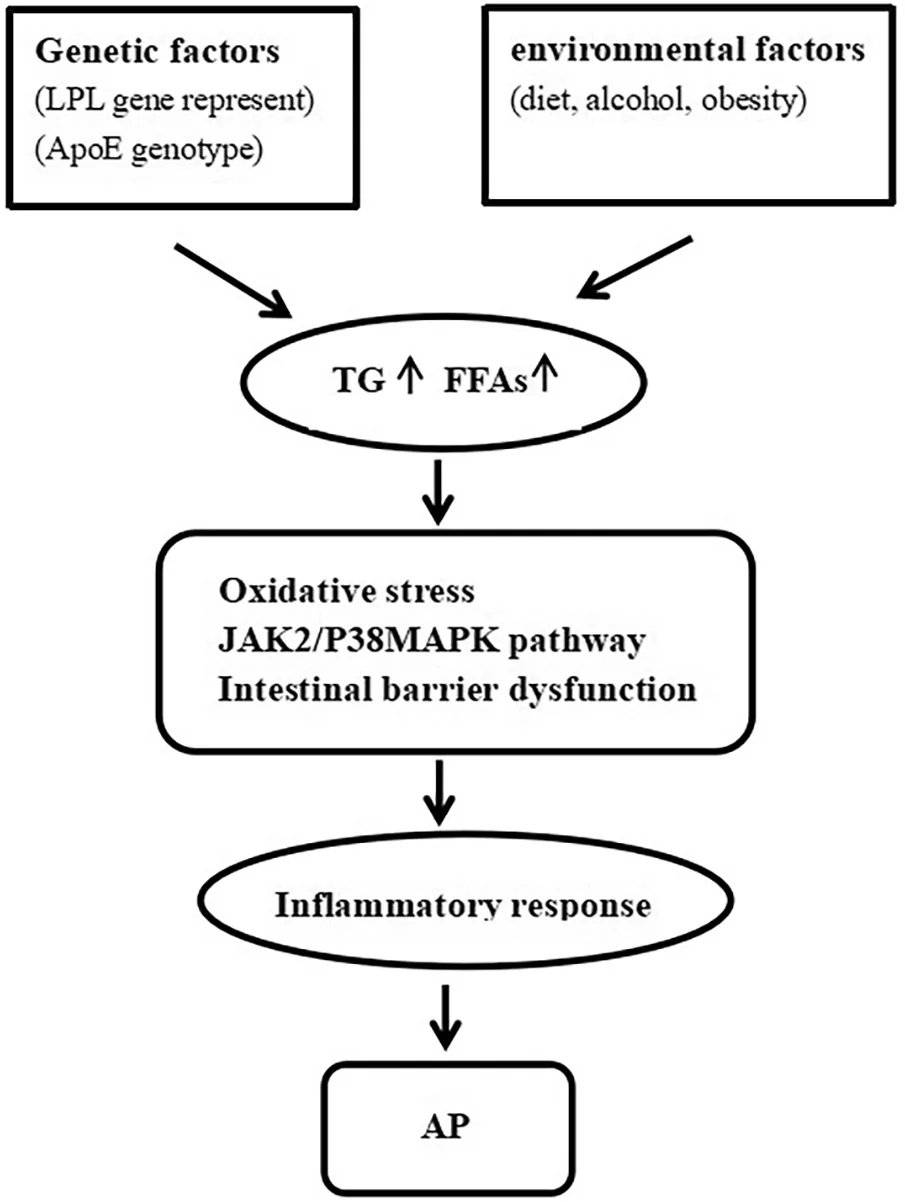
Schematic diagram of the pathophysiology of HTG-AP. Genetic predisposition and environmental triggers (such as high-fat diet, alcohol consumption, and obesity) lead to severe hypertriglyceridemia. High triglyceride levels lead to the excessive release of free fatty acids (FFAs), triggering oxidative stress, the activation of the JAK2/P38MAPK signaling pathway, and intestinal barrier dysfunction, ultimately resulting in pancreatic acinar cell necrosis and systemic inflammation.

The core mechanism by which hypertriglyceridemia induces acute pancreatitis involves direct lipotoxic injury to pancreatic cells and the amplification of systemic inflammatory responses driven by lipid metabolism disorders. Research indicates that in the setting of hypertriglyceridemia, pancreatic lipase hydrolyzes excessive TG to generate a massive burden of free fatty acids (FFAs). These FFAs induce acinar cell necrosis by disrupting pancreatic cell membrane integrity, triggering calcium overload, and activating oxidative stress pathways (12). Animal studies demonstrate that in rat models induced by a high-fat diet, reactive oxygen species (ROS) levels in pancreatic tissue are significantly elevated; malondialdehyde (MDA) content increases 2.3-fold compared to normal diet controls, while superoxide dismutase (SOD) activity decreases by 40%, underscoring the pivotal role of oxidative stress in HTG-AP pathogenesis (12). Furthermore, activation of the JAK2 signaling pathway exacerbates pancreatic injury by regulating necrosis and inflammatory responses. In models of AP induced by a high-fat diet combined with caerulein, the JAK2 inhibitor AG490 reduced the area of pancreatic necrosis by 58% and lowered serum TNF-α levels by 62% (13). Intestinal barrier dysfunction constitutes another critical pathological link in HLAP: expression of glutathione S-transferase pi (GSTpi) in colonic tissue is reduced by 60% in HTG-AP patients compared to those with other forms of AP, leading to increased intestinal permeability, endotoxin translocation into the bloodstream, and the subsequent triggering of a systemic inflammatory response (14). Metabolomic studies further reveal significant abnormalities in serum metabolites such as phosphatidylethanolamine (PE) and TG in HTG-AP patients. Notably, PE (18:0/22:6) exhibits a diagnostic area under the curve (AUC) of 0.962, suggesting a tight correlation between lipid metabolism disorders and inflammatory responses (15). Schematic diagram of the pathophysiology of HTG-AP (Figure 1).

## Clinical manifestations and diagnosis of HTG-AP

The clinical symptoms of HTG-AP are generally non-specific, yet certain characteristic features distinguish it from other etiologies. Typical presentations include persistent upper abdominal pain (95%), nausea and vomiting (80%), and fever (60%); however, a subset of patients may present without significant abdominal pain, manifesting solely as abdominal distension or dyspnea (5, 10). The severity of HTG-AP is classified into three grades: mild, moderately severe, and severe. The core criteria for grading are the duration of organ failure and the presence of local or systemic complications, with persistent organ failure lasting >48 h defining severe disease. Mild acute pancreatitis (MAP): Characterized by the absence of organ failure and no local or systemic complications, with an extremely low mortality rate. Moderately severe acute pancreatitis (MSAP): Associated with transient (≤48 h) organ failure, or local/systemic complications, with a mortality rate of <5%. Severe acute pancreatitis (SAP): Associated with persistent (>48 h) organ failure, with a mortality rate as high as 36%∼50%. Compared to AP of other causes, HTG-AP patients are more prone to multiple organ dysfunction: 44.9% develop respiratory failure and 28.6% suffer from acute kidney injury (AKI), with incidence rates for these complications being twofold higher than those observed in biliary AP (3). Symptoms in pregnant women with HTG-AP are particularly fulminant, often presenting as acute respiratory distress syndrome (ARDS) requiring mechanical ventilation support (16). Furthermore, HTG-AP patients may exhibit cutaneous manifestations, such as eruptive xanthomas (yellowish papules predominantly on the trunk and extensor surfaces of the limbs), which indicate TG levels exceeding 4,000 mg/dl (17). Notably, C-reactive protein (CRP) levels in HTG-AP patients can exceed 200 mg/L and show a positive correlation with disease severity (3).

Imaging modalities play a pivotal role in the diagnosis and severity assessment of HTG-AP. Abdominal computed tomography (CT) is the preferred initial modality, capable of visualizing pancreatic edema, necrosis, and peripancreatic exudates; studies indicate that the incidence of pancreatic necrosis in HTG-AP patients reaches 33.05%, with 25.57% presenting as mixed necrosis (18). Magnetic resonance imaging (MRI) offers superior delineation of pancreatic parenchyma and peripancreatic lesions, demonstrating particular advantage in assessing infected necrotic tissue (1). Although ultrasonography is often limited by bowel gas interference, it remains valuable for excluding biliary calculi and evaluating pancreatic enlargement and peripancreatic fluid collections (19). Notably, HTG-AP exhibits distinct imaging characteristics: the proportion of diffuse pancreatic involvement (Type III) reaches 23.75%, and patients with this pattern present with more severe disease, evidenced by a higher mean Modified CT Severity Index (MCTSI) score of 7 (1). Furthermore, the CT Severity Index (CTSI) serves as a robust tool for stratifying HTG-AP severity, showing a significant correlation with both length of hospital stay and complication rates (*r* = 0.72, *P* < 0.001) (20). In recent years, machine learning models integrating CT radiomic features with clinical indicators have demonstrated enhanced accuracy in predicting organ failure in HLAP, achieving an AUC of 0.915 (21).

Significant advancements in biomarker research for HTG-AP have provided novel tools for early diagnosis and severity assessment. Metabolomic studies have revealed significant abnormalities in serum lipid metabolites among HTG-AP patients, particularly PE(O-16:0/20:4) and TG(48:3/FA18:1); notably, PE(18:0/22:6) has demonstrated a diagnostic AUC of 0.962 (15). Regarding inflammatory markers, D-dimer levels are markedly elevated in HTG-AP patients (mean: 2.5 mg/L vs. 1.2 mg/L in non-HTG-AP) and show a strong positive correlation with disease severity (*r* = 0.68, *P* < 0.001) (22). Furthermore, the combined detection of the neutrophil-to-lymphocyte ratio (NLR), platelet-to-lymphocyte ratio (PLR), and triglyceride-to-high-density lipoprotein cholesterol ratio (TG/HDL-C) enhances diagnostic accuracy, yielding a combined AUC of 0.898 (23, 24). At the molecular level, miR-214-3p is upregulated in the pancreatic and renal tissues of HTG-AP patients, where it exacerbates inflammatory responses by suppressing the PTEN/Akt pathway (25). Notably, serum levels of lipoprotein-associated phospholipase A2 (PLA2G7) are significantly elevated in HTG-AP patients and correlate positively with triglyceride levels (*r* = 0.75, *P* < 0.001), it not only reflects the severity of systemic inflammation but also provides crucial reference for the early warning of multiple organ failure. Combining it with clinical scoring systems helps clinicians identify high-risk patients earlier and optimize treatment strategies, making it a potential indicator for disease monitoring (26). Table 1 provides a detailed summary of the diagnostic approaches for HTG-AP, including clinical criteria, TG thresholds, laboratory tests, imaging modalities, and biomarkers, along with their respective advantages and limitations.

**Table 1:** Diagnostic approaches for hypertriglyceridemia-induced acute pancreatitis (HTG-AP).

Category	Diagnostic modality	Key findings/criteria	Advantages/limitations
Clinical criteria	Typical symptoms: persistent upper abdominal pain (95%), nausea/vomiting (80%), fever (60%) Severity grading: MAP (no organ failure, no complications), MSAP (transient organ failure ≤48 h or complications; mortality <5%), SAP (persistent organ failure >48 h; mortality 36%–50%) Cutaneous: eruptive xanthomas indicate TG >4,000 mg/dl CRP >200 mg/L correlates with severity	Readily available, non-invasive, guides severity assessment and triage Widely used in clinical practice and clinical trials	Non-specific symptoms; cannot confirm HTG-AP etiology alone CRP elevation is not specific to HTG-AP
TG thresholds	Diagnostic threshold: serum TG ≥11.3 mmol/L Alternative: TG 5.65–11.3 mmol/L with turbid, chylous serum after excluding other causes (gallstones, alcohol)	Simple, objective, widely available laboratory test Provides clear diagnostic cutoff	TG levels may normalize by hospital presentation Does not distinguish HTG-AP from other AP etiologies
Laboratory tests	D-dimer: markedly elevated (mean 2.5 vs. 1.2 mg/L); *r* = 0.68 with severity (*P* < 0.001) Combined NLR + PLR + TG/HDL-C: AUC = 0.898 CRP >200 mg/L shows positive correlation with disease severity	Widely available, supports diagnosis and severity stratification Combined markers improve diagnostic accuracy	Non-specific; may be elevated in other AP types No single marker is definitive for HTG-AP etiology
Imaging	Abdominal CT (preferred): visualizes pancreatic edema, necrosis (rate 33.05%), peripancreatic exudates; CTSI correlates with LOS and complications (*r* = 0.72) MRI: superior for delineating parenchyma and infected necrotic tissue Ultrasound: limited by bowel gas; valuable for excluding biliary calculi CT radiomics + machine learning: AUC = 0.915 for predicting organ failure	CT visualizes complications and guides management MRI excellent for infected necrosis assessment AI models show high predictive accuracy	CT involves ionizing radiation MRI costly and less accessible US operator-dependent; limited by bowel gas
Biomarkers	PE(18:0/22:6): diagnostic AUC = 0.962 miR-214-3p: upregulated in pancreatic/renal tissue; exacerbates inflammation via PTEN/Akt pathway PLA2G7 (Lp-PLA2): elevated; correlates with TG levels (*r* = 0.75, *P* < 0.001) TMAO: aggravates inflammation via TLR/p65 pathway Ceramide: elevated; correlates with pancreatic injury severity	High diagnostic accuracy (AUC up to 0.962) Potential for early detection and severity prediction Metabolomic profiling AUC up to 1.0	Mostly research-stage; not yet widely available clinically Expensive; requires specialized equipment Not yet incorporated into clinical guidelines

## Therapeutic strategies for HTG-AP

Pharmacological management of HTG-AP centers on the rapid reduction of TG levels and the suppression of inflammatory responses. The combination of insulin and heparinrepresents a conventional lipid-lowering regimen: insulin activates lipoprotein lipase (LPL) to accelerate TG hydrolysis, while heparin facilitates the release of LPL from the endothelial surface; together, this synergistic approach can reduce TG levels by 50% within 48 h (27). Fibrates (e.g., fenofibrate) lower TG levels by activating peroxisome proliferator-activated receptor alpha (PPARα), although clinicians must remain vigilant regarding the potential risk of rhabdomyolysis (28). Omega-3 fatty acids lower TG levels by reducing TG synthesis and secretion. Niacin exerts broad-spectrum lipid-modulating effects by activating niacin receptors to reduce lipolysis, thereby lowering both TG and cholesterol levels. However, due to adverse effects such as skin flushing and increased hepatic gluconeogenesis, it is rarely used in current clinical practice.

Recently, novel lipid-lowering agents such as volanesorsen, olezarsen, plozasiran, an antisense oligonucleotide targeting apolipoprotein C-III (APOC3), have garnered significant attention; volanesorsen has been shown to reduce TG levels by 60% and decrease the frequency of pancreatitis recurrence (29). Olezarsen can specifically bind to APOC3 mRNA, preventing the production of APOC3, thereby relieving the inhibition on LPL and promoting the clearance of triglyceride-rich lipoproteins in the blood. Plozasiran is a small interfering RNA (siRNA) drug targeting APOC3. It degrades the target mRNA to block the synthesis of APOC3 at the source, thus relieving its inhibition on LPL and accelerating the clearance of TG. Furthermore, traditional Chinese medicine formulations, such as Da Cheng Qi Tang, have demonstrated efficacy in ameliorating HTG-AP symptoms by modulating gut microbiota and inhibiting inflammatory responses; when combined with standard care, this regimen has improved the total effective rate from 75% to 95% (30). Notably, pharmacological strategies must be tailored to individual patient profiles: for pregnant women with HTG-AP, statins are contraindicated, and the preferred approach involves insulin therapy combined with therapeutic plasma exchange (5).

Nutritional support for HTG-AP must strike a delicate balance between strict lipid control and meeting metabolic demands. During the acute phase, a fat-free or very low-fat diet is imperative, transitioning gradually to a low-fat regimen (fat intake <20 g/day) once clinical stability is achieved (31). Early initiation of enteral nutrition (EN) within 48 h is strongly recommended; administering low-fat formulations via a nasojejunal tube has been shown to significantly reduce the incidence of infectious complications compared to total parenteral nutrition (TPN) (20% vs. 35%) (2). For patients intolerant to EN, parenteral nutrition utilizing lipid emulsions, particularly those rich in medium-chain triglycerides (MCTs), may be employed, necessitating rigorous monitoring of serum TG levels (32). Furthermore, probiotics have emerged as a beneficial adjunct by modulating gut microbiota to improve lipid metabolism, with animal studies demonstrating a 30% reduction in serum TG levels (33). Nutritional management must also address micronutrient deficiencies; notably, vitamin D deficiency is prevalent in up to 60% of HTG-AP patients, warranting timely supplementation to bolster immune function (34). Crucially, nutritional support for pregnant women with HTG-AP requires a personalized approach developed under the guidance of a dietitian to simultaneously meet maternal therapeutic goals and fetal developmental needs (5).

Surgical intervention in HTG-AP is primarily reserved for managing complications such as infected pancreatic necrosis and pseudocysts. Percutaneous catheter drainage (PCD) serves as the first-line treatment for infected necrosis, achieving a success rate of 85% while significantly reducing procedure-related complications (35). For patients unresponsive to PCD, endoscopic necrosectomy offers a minimally invasive alternative to traditional open surgery, shortening the hospital stay by approximately 10 days (36). The role of plasma exchange (PE) in HTG-AP remains controversial: although it rapidly reduces TG levels by up to 78.8%, some studies indicate no significant improvement in overall clinical outcomes (37, 38). Continuous renal replacement therapy (CRRT) is indicated for HTG-AP patients complicated by multiple organ dysfunction, as it effectively clears inflammatory mediators and improves organ function (39). Timing of intervention is critical; drainage or debridement for infected necrosis should ideally be delayed until at least 4 weeks after onset to minimize the risk of hemorrhage (32). Regarding efficacy evaluation, the Modified CT Severity Index (MCTSI) is a valuable tool for assessing surgical outcomes, with a post-operative reduction of ≥3 points indicating therapeutic success (20).

## Technological advances in HTG-AP

Molecular biology research into HTG-AP has elucidated several critical signaling pathways and molecular targets. The HIF-1α–PPARγ–mTORC1 axis plays a pivotal role in regulating autophagy in HTG-AP: hyperlipidemia induces upregulation of hypoxia-inducible factor-1α (HIF-1α) and suppression of peroxisome proliferator-activated receptor gamma (PPARα), subsequently activating mechanistic target of rapamycin complex 1 (mTORC1), which promotes aberrant autophagy and exacerbates inflammatory responses (37). Animal studies demonstrate that inhibition of HIF-1α reduces pancreatic inflammation scores by 50% in HTG-AP models (37). Furthermore, MRG15 promotes pancreatic acinar cell apoptosis by degrading translation elongation factor Tu, mitochondrial (TUFM) and thereby inhibiting mitophagy; its expression is elevated 2.5-fold in pancreatic tissues of HTG-AP patients compared to normal controls (40). The interplay between the gut microbiota and HTG-AP has also garnered significant attention: HTG-AP patients exhibit a decreased abundance of Bacteroidetes and an increased abundance of Firmicutes, while microbial metabolites such as trimethylamine N-oxide (TMAO) aggravate inflammation via the TLR/p65 pathway (41). Additionally, metabolomic studies reveal significant dysregulation of sphingolipid metabolism in HTG-AP patients, with elevated ceramide levels showing a positive correlation with the severity of pancreatic injury (26).

The application of precision medicine in HTG-AP is primarily manifested through individualized diagnosis and tailored therapeutic strategies. Genetic testing enables the identification of underlying hereditary etiologies, such as defects in LPL or APOC2, thereby providing a critical basis for selecting optimal treatment regimens (42). For instance, patients harboring LPL mutations often exhibit suboptimal responses to conventional lipid-lowering pharmacotherapy, necessitating alternative interventions such as gene therapy or therapeutic plasma exchange (26, 43). Metabolomic profiling facilitates early diagnosis, with constructed diagnostic models demonstrating an AUC of up to 1.0 (26). Furthermore, machine learning algorithms that integrate clinical parameters with radiological features can effectively predict the risk of organ failure in HTG-AP, achieving an AUC of 0.883 (44). In terms of precision therapeutics, lipid-lowering protocols can be personalized based on individual genetic backgrounds and metabolic profiles; for example, HTG-AP patients with comorbid diabetes are prioritized for a combination of insulin and glucagon-like peptide-1 (GLP-1) receptor agonists (11). Notably, the successful implementation of precision medicine requires a robust multidisciplinary team approach, encompassing geneticists, dietitians, and clinicians (42).

Personalized therapy will constitute the cornerstone of future HTG-AP management. Treatment selection guided by genetic profiling is paramount: for instance, patients harboring LPL mutations may benefit from gene therapy, whereas those with APOC3 mutations are ideal candidates for volanesorsen (29). Additionally, gut microbiota modulation is emerging as a novel therapeutic avenue; probiotics have been shown to improve lipid metabolism, with animal studies indicating a 30% reduction in serum TG levels (33). Metabolomics-guided nutritional interventions offer another layer of personalization, enabling the formulation of tailored dietary plans based on individual metabolic profiles, such as reducing saturated fatty acid intake in patients with elevated PE levels (15). Crucially, artificial intelligence models capable of integrating clinical data with genetic characteristics can predict therapeutic responses, thereby optimizing treatment regimens for each patient (44). Table 2 comprehensively outlines the current and emerging therapeutic strategies for HTG-AP, detailing the level of evidence and major limitations for each approach.

**Table 2:** Current and emerging therapeutic strategies for hypertriglyceridemia-induced acute pancreatitis (HTG-AP).

Therapeutic strategy	Mechanism of action	Efficacy/evidence	Level of evidence	Major limitations	Special considerations
Supportive care (Nutritional support)	Early enteral nutrition (EN) within 48 h via nasojejunal tube; low-fat diet (<20 g/day) once stable; fat-free/very low-fat diet in acute phase Probiotics modulate gut microbiota to improve lipid metabolism	EN reduces infectious complications vs. TPN (20% vs. 35%) Probiotics: 30% reduction in serum TG (animal studies) Combined with standard care, total effective rate improved from 75% to 95%	Moderate	Risk of malnutrition if lipid restriction too strict TPN associated with higher infection rates Vitamin D deficiency prevalent in up to 60% of patients	Pregnant women require personalized nutritional plans under dietitian guidance
Insulin	Activates lipoprotein lipase (LPL) to accelerate TG hydrolysis	Combined with heparin, reduces TG by ∼50% within 48 h	Moderate	Hypoglycemia risk Requires careful blood glucose monitoring	Preferred approach in pregnancy (statins contraindicated)
Heparin	Facilitates release of LPL from endothelial surface; synergizes with insulin	Synergistic TG-lowering effect when combined with insulin ∼50% TG reduction within 48 h	Low–Moderate	Bleeding risk Mechanism-based rationale lacks RCT confirmation	Must be used in conjunction with insulin for optimal effect
Plasma exchange (Plasmapheresis)	Direct removal of TG-rich lipoproteins from circulation	Rapidly reduces TG by 69.57%–78.8% Some studies show no significant improvement in overall clinical outcomes	Low–Moderate	Controversial efficacy on clinical outcomes Costly; invasive procedure Not consistently improved outcomes in some studies	Preferred in pregnancy when combined with insulin Timing and frequency require careful balance of maternal/fetal safety
CRRT (Continuous renal replacement therapy)	Clears inflammatory mediators; improves organ function in MODS	Effective for managing MODS complications Improves organ function in multi-organ failure	Low	Not a primary TG-lowering modality Indicated mainly for MODS complications	Reserved for HTG-AP patients with multiple organ dysfunction
Fibrates (e.g., fenofibrate)	Activates PPARα to lower TG synthesis and increase TG clearance	Effective oral lipid-lowering agent Combined with standard care, total effective rate 95%	Moderate	Risk of rhabdomyolysis Contraindicated in pregnancy	Must monitor for muscle toxicity; avoid in pregnant patients
Omega-3 fatty acids	Reduces TG synthesis and secretion; broad-spectrum lipid modulation	Lowers TG levels Adjunctive therapy with modest effect	Low–Moderate	Modest TG-lowering effect Not sufficient as monotherapy for severe HTG-AP	Best used as adjunctive therapy rather than standalone treatment
APOC3 inhibitors (volanesorsen, olezarsen, plozasiran)	Volanesorsen: antisense oligonucleotide targeting APOC3 mRNA Olezarsen: binds APOC3 mRNA, preventing APOC3 production Plozasiran: siRNA targeting APOC3; degrades target mRNA	Volanesorsen reduces TG by 60% and decreases pancreatitis recurrence Specifically relieves LPL inhibition, promoting TG clearance	Emerging	Long-term safety not established Limited clinical data Expensive; novel agents with restricted availability	Ideal candidates: patients with APOC3 mutations Part of precision medicine approach
Gene therapy	Corrects underlying genetic defects (e.g., LPL gene mutations) Replaces or repairs defective LPL gene	Potential curative approach for monogenic HTG-AP LPL mutation carriers often suboptimal response to conventional therapy	Emerging (preclinical)	Not yet clinically available Limited to monogenic forms Technical and safety challenges	Patients with LPL mutations may benefit most May require alternative interventions (plasma exchange) in the interim
Percutaneous catheter drainage (PCD)	Drainage of infected pancreatic necrosis and pseudocysts	First-line treatment for infected necrosis; success rate 85% Significantly reduces procedure-related complications	Moderate	Should be delayed until ≥4 weeks after onset to minimize hemorrhage risk Not a TG-lowering strategy	For patients unresponsive to PCD, endoscopic necrosectomy is an alternative MCTSI reduction ≥3 points indicates therapeutic success

## Controversies and challenges in HTG-AP

Although significant progress has been made in the treatment of HTG-AP, many challenges remain to be addressed. Selecting optimal therapeutic regimens for HTG-AP presents significant challenges, particularly concerning lipid-lowering strategies and the timing of surgical interventions. The comparative efficacy of therapeutic plasma exchange (TPE) versus insulin-heparin combination therapy remains contentious: while some studies demonstrate that TPE can rapidly reduce TG levels by up to 85%, it has not consistently translated into improved clinical outcomes (38); conversely, insulin-heparin therapy, though slower in lowering lipids, offers a more cost-effective alternative. Managing HTG-AP during pregnancy poses additional complexities, necessitating a delicate balance between maternal and fetal safety, particularly regarding the timing and frequency of TPE (5). Future randomized controlled trials are essential to clearly delineate the indications and comparative efficacy of these diverse therapeutic modalities.

## Conclusion

HTG-AP has emerged as the second most common etiology of acute pancreatitis, characterized by a rising global incidence and a younger demographic onset. The pathogenesis of this disease is multifactorial, involving a complex interplay between genetic susceptibility and environmental factors. Although conventional management primarily focuses on rapidly lowering triglyceride levels through insulin-heparin therapy or apheresis (plasmapheresis), recent advances in precision medicine have introduced novel therapeutic targets (such as volanesorsen, olezarsen, and plozasiran), marking a significant paradigm shift in treatment. Furthermore, the integration of metabolomics and machine learning offers promising tools for early diagnosis and risk stratification. Future research must prioritize the optimization of personalized therapeutic strategies and the construction of comprehensive prevention systems to mitigate the substantial healthcare burden imposed by HTG-AP.
